# Intermittent Fasting Enhanced the Cognitive Function in Older Adults with Mild Cognitive Impairment by Inducing Biochemical and Metabolic changes: A 3-Year Progressive Study

**DOI:** 10.3390/nu12092644

**Published:** 2020-08-30

**Authors:** Theng Choon Ooi, Asheila Meramat, Nor Fadilah Rajab, Suzana Shahar, Intan Safinar Ismail, Amalina Ahmad Azam, Razinah Sharif

**Affiliations:** 1 Centre for Healthy Aging and Wellness, Faculty of Health Science, Universiti Kebangsaan Malaysia, Jalan Raja Muda Abdul Aziz, Kuala Lumpur 50300, Malaysia; ooithengchoon@ukm.edu.my (T.C.O.); nfadilah@ukm.edu.my (N.F.R.); suzana.shahar@ukm.edu.my (S.S.); 2 Faculty of Health Sciences, Universiti Sultan Zainal Abidin, Gong Badak Campus, Kuala Nerus, Terengganu 21300, Malaysia; asheilameramat@unisza.edu.my; 3 Laboratory of Natural Products, Institute of Bioscience, Universiti Putra Malaysia, Serdang, Selangor 43400, Malaysia; safinar@upm.edu.my (I.S.I.); amalina_azam@hotmail.com (A.A.A.); 4 Biocompatibility Laboratory, Centre for Research and Instrumentation, Universiti Kebangsaan Malaysia, Bangi 43600 UKM, Selangor, Malaysia

**Keywords:** DNA damage, intermittent fasting, metabolomics, mild cognitive impairment, oxidative stress, inflammation, older adults

## Abstract

Intermittent fasting (IF) refers to various dietary regimens that cycle between a period of non-fasting and a period of total fasting. This study aimed to determine the effects of IF on cognitive function among elderly individuals who practice IF who have mild cognitive impairment (MCI). A total of 99 elderly subjects with MCI of Malay ethnicity without any terminal illness were recruited from a larger cohort study, LRGS TUA. The subjects were divided into three groups, comprising those who were regularly practicing IF (r-IF), irregularly practicing IF (i-IF), and non-fasters (n-IF). Upon 36 months of follow-up, more MCI subjects in the r-IF group reverted to successful aging with no cognitive impairment and diseases (24.3%) compared to those in i-IF (14.2%) and n-IF groups (3.7%). The r-IF group’s subjects exhibited significant increment in superoxide dismutase (SOD) activity and reduction in body weight, levels of insulin, fasting blood glucose, malondialdehyde (MDA), C-reactive protein (CRP), and DNA damage. Moreover, metabolomics analysis showed that IF may modulate cognitive function via various metabolite pathways, including the synthesis and degradation of ketone bodies, butanoate metabolism, pyruvate metabolism, and glycolysis and gluconeogenesis pathways. Overall, the MCI-afflicted older adults who practiced IF regularly had better cognitive scores and reverted to better cognitive function at 36 months follow-up.

## 1. Introduction

Neurodegenerative diseases, a heterogeneous group of disorders, are characterized by slow progressive loss of neurons [1]. Although the precise etiology underlying neurodegeneration has not been fully elucidated, oxidative stress has been suggested as one of the contributing factors of various neurodegenerative diseases and accelerated aging [2,3,4,5]. Levels of oxidative damage correlate significantly with the neurodegenerative impairment in various populations. For these reasons, there is a great interest among researchers in finding ways to protect against oxidative damage and potentially treat neurodegenerative diseases, especially among individuals with mild cognitive impairment (MCI). MCI is a pre-stage for dementia, and it is known to be reversible. Previous research has reported that MCI is associated with impairment of glucose metabolism in the brain, dietary composition, and caloric intake [6].

Dietary approaches are suggested as more viable and non-invasive ways to prevent MCI incidence and to promote the reversion of MCI among the general public. Caloric restriction (CR) is one of the dietary regimens that have been shown to produce positive health effects. However, several alternative hypotheses argued that CR could cause damage to protein, lipids, and nucleic acids through the accumulation of reactive oxygen species (ROS) or reactive nitrogen species (RNS) [7]. Moreover, the compliance to long-term hypocaloric diet is inadequate, and other approaches more acceptable from the older adult perspective are needed. Thus, intermittent fasting (IF) is another approach that represents an alternative and more physiological way to prevent the deleterious effect of chronic excess of food intake [8].

IF is an umbrella term referring to various dietary regimens that cycle between a period of non-fasting and a period (long or short) of total fasting [9]. IF was intended as an alternative regimen that takes advantage of CR benefits, without eliciting any negative side effects from severe CR that causes malnourishment. Several studies support the idea that IF and CR activate similar biological mechanisms [10]. Interestingly, IF also reduces body weight the same as CR, but the difference is that CR produces loss of muscle mass and adipose tissue whereas IF reduces the adipose tissues whilst preserving muscle mass both in human and animal models [11]. Many scientific studies have been carried out, finding that IF gives beneficial health improvement by prolonging the lifespan and prevention of other chronic diseases including cardiovascular diseases, different forms of cancer, diabetes, and renal diseases [12,13].

Although previous studies have demonstrated the beneficial effects of IF with or without CR amongst an older population, most of these studies focused on healthy and obese people with no or limited MCI only. Fasting CR conducted among older adult men for 3 months via a clinical trial using a combination regime of CR with 2 days/weeks of Muslim sunnah fasting showed that CR with IF could improve metabolic parameters and quality of life, and alleviate the depression among these older subjects [12]. IF was also reported as one of the factors for reducing the risk of cognitive impairment among older adults in a large cohort conducted in Malaysia [14]. For these reasons, there is a need to identify the health benefits of IF among older adults with MCI. Hence, this study aims to investigate the effects of IF on biochemical profile, cognitive function, oxidative stress, genome health, and inflammatory responses among older adults with MCI. Besides this, this study also aims to identify the neuroprotective metabolites among older adults with MCI who practice IF since the human metabolomics data on this aspect remain unavailable.

## 2. Materials and Methods

### 2.1. Sample Size Calculation

The sample size was calculated using G*power software. F-test (analysis of covariance (ANCOVA): fixed effects, omnibus, one-way) was used to calculate the sample size. In this study, the effect of medium size (f) and error probability (α) were set to 0.40 and 0.05, respectively, while power (1-β) was 0.80. 

### 2.2. Subject Recruitment

This study is a part of the longitudinal study on the neuroprotective model for healthy longevity (LRGS TUA) involving Malaysian older adults from four states in Malaysia with the highest prevalence of older adults aged 60 years and above [15]. Only Muslim subjects with MCI adhering to Petersen’s criteria were recruited in this study [16]. Subjects were recruited for baseline analysis from May 2012 to February 2013 and were followed up for 36 months. Out of the 967 Malay subjects, only 118 subjects fulfilled the criteria. However, 19 subjects could not be followed up at 36 months because they could not be contacted, died, moved, could not complete all the study tests, or refused to follow this study anymore. Meanwhile, a total of 99 subjects met all the criteria of the study and agreed to participate in this study. Subjects who agreed to participate in this study were given a complete description of the study procedure as well as signing the consent form. This study was conducted in accordance with the Declaration of Helsinki and was approved by the Medical Research and Ethics Committee of University Kebangsaan Malaysia (UKM 1.5.3.5/244/NN-060-2013). 

### 2.3. Intermittent Fasting Grouping

In this study, subjects were grouped into three groups according to their IF status (regularly practicing IF, r-IF; irregularly practicing IF, i-IF; or not practicing IF, n-IF). Subjects in the r-IF group had fasted for 12 months before baseline recruitment and continued to practice IF for 36 months until follow-up. IF was practiced by fasting on Monday and Thursday every week (Sunnah fasting) beginning from sunrise to sunset. Drinking was not allowed during fasting. Subjects in the i-IF group had fasted for 12 months before baseline recruitment but stopped practicing IF after baseline until the follow-up or vice-versa. Subjects that did not practice IF before or after both baseline recruitment and 36 months of follow-up were categorized into the n-IF group. Following the grouping criteria, 37, 35, and 27 subjects were grouped under the r-IF group, i-IF, group and n-IF group, respectively. Then, 15 subjects were randomly selected from each IF group (a total of 45 subjects) and were subjected to metabolomics analysis.

### 2.4. Socio-Demographic Data

A structured interview was administered to obtain the socio-demographic data, which included age, gender, marital status, living status, years of education, and smoking habits [17]. 

### 2.5. Body Composition and Blood Pressure Measurement

The height, body weight, circumference of waist and hip, and blood pressure were measured as previously described [17]. Height was measured without shoes, using a SECA 206 portable body meter (Seca, Hamburg, Germany), while body weight was measured in light clothing without shoes, using a Tanita digital lithium weighing scale (Tanita, Tokyo, Japan). The readings for height and weight were taken to the nearest 0.1 cm and 0.1 kg, respectively. Then, the circumference of the waist and hip was measured using a Lufkin tape. Meanwhile, the subject’s systolic and diastolic blood pressure were measured using a calibrated digital automatic blood pressure monitor (OMRON, Kyoto, Japan). The blood pressure of each subject was measured twice to obtain an average reading.

### 2.6. Assessment of Cognitive Function

Cognitive status was evaluated using five types of cognitive test that assessed different domains of the human brain, including the Malay version of the Mini Mental Examination State (MMSE), Malay version of Montreal Cognitive Assessment (MoCA), Rey Auditory Verbal Learning Test (RAVLT), Digit Span Test, and Digit Symbol, as previously described [17]. Validated Malay versions of cognitive tests were employed in this study to ensure these tests fit into the Malaysian norm to avoid any misinterpretation and bias.

### 2.7. Cognitive Aging Grouping

Subjects were divided into three groups according to the aging criteria: (a) successful aging (i) free from six chronic diseases including high blood pressure, diabetes mellitus, cancer, chronic lung disease, stroke, and heart disease, (ii) good functional ability (instrumental activities of daily living (IADL) ≥ 14 and activities of daily living (ADL) ≥ 6), (iii) no depression (geriatric depression scale ≤ 4), (iv) good cognitive function (MMSE ≥ 22), (v) good quality of life and good health status; (b) normal aging, (i) no dementia, (ii) moderate score for cognitive performance (higher from MCI group and lower score from the successful aging group); (c) MCI, (i) global cognitive function is preserved (MMSE ≥19), (ii) none or minimal functional limitations (IADL ≥ 9), (iii) no restriction on basic daily living activities (ADL = 6) or subjective memory complaints by the respondent or guardian, (iv) objectively impaired memory characterized by poor performance in at least one of the cognitive test (T5 trial RAVLT score ≥ 34 or Digit Span Scale score ≤ 4) [18,19].

### 2.8. Biochemical Analyses

Fasting venous blood was collected at both baseline and 36 months follow-up by a certified phlebotomist for biochemical analyses. Blood collected using ethylenediaminetetraacetic acid (EDTA) tubes and sodium fluoride tube was sent to a certified medical laboratory to determine the lipid profile and fasting blood glucose level of each subject. On the other hand, whole blood collected using heparin tubes was used for DNA damage analysis while blood plasma was kept at −80°C and was used for the analysis of malondialdehyde (MDA), superoxide dismutase (SOD) activity, inflammatory markers, and insulin. 

### 2.9. DNA Damage Analysis

DNA damage was determined using alkaline comet assay as previously described [20]. Briefly, 50 μL of whole blood were embedded in 70 μL of agarose gel (Sigma, USA) on frosted slides prior to lysis in denaturing buffer and electrophoresis (300 mA, 25 V). After electrophoresis, the slides were stained with ethidium bromide (Sigma, St. Louis, MO, USA). The comet image was captured under the Olympus BX51 fluorescence microscope (Olympus, Tokyo, Japan) and the data were analyzed using the Comet Assay III single-cell gel image analysis program (Perspective Instruments Ltd, Suffolk, UK). To determine the DNA damage, percentage tail DNA (the percentage of total nuclear DNA that has migrated to the tail) (TD) and tail moment (%Tail DNA × length) (TM) were quantified. Each slide was analyzed in duplicate, representing each subject, and 50 comets per slide were scored for each subject. 

### 2.10. Malondialdehyde Analysis

MDA level was analyzed as previously described by Stocks et al. (1974) [21]. The absorbance of MDA was measured using a spectrophotometer at 532 nm wavelength after reaction with thiobarbituric acid under 30 min of heating. 

### 2.11. Superoxide Dismutase Analysis

Briefly, 20 μL of plasma was mixed with 250 μL of substrate mixture (27 mL of phosphate buffer (pH 7.8), 1.0 mL of methionine, 1 mL of NBT.2HCl, 250 μL of riboflavin, and 0.75 mL of Triton-X) on 96-well plates. The mixture was placed inside an aluminum box for 7 min to allow the reaction with Sylvania arolux fluorescent lamp (18 watts). Absorbance change was measured using a spectrophotometer and presented relative to assay control. 

### 2.12. Inflammatory Marker and Insulin Analysis

Plasma C-reactive protein (CRP) (catalog no. E-EL-H0043, Elabscience) and insulin (catalog no. E-EL-H5439, Elabscience) level was measured in duplicate using commercially available enzyme-linked immunosorbent assay (ELISA) kits (Elabscience, Wuhan, China) following the manufacturer’s guidelines.

### 2.13. ^1^H Nuclear Magnetic Resonance Spectroscopic Analysis

Untargeted metabolite profiling and identification were analyzed by nuclear magnetic resonance (NMR) spectroscopy. All plasma samples were analyzed by ^1^H NMR spectroscopy at 700 MHz using a Bruker Avance II 700 spectrometer (Bruker Biospin, Germany) operating at 300 K following the method previously described [22]. The ^1^H NMR spectra were baseline-corrected (flattened) in Chenomx before data processing. Spectral resonance in plasma from trimethylsilyl propionic acid-d_4_ sodium salt (TSP) (0.0 ppm) and residual water peak (4.72–5.00 ppm) was excluded from analyses. The spectrum ranging from 10.0 to 0.5 ppm was divided into 2325 integral segments of equal length (0.04 ppm). The area under the spectrum was calculated for each segmented region and expressed as an integral value. To reduce the dimensionality and mitigate peak misalignment, we employed a dynamic programming-based adaptive binning technique using a minimum and maximum distance between peaks in a single bin of 0.001 and 0.04 ppm, respectively. Bin boundaries were then manually adjusted to further mitigate peak misalignment, and to keep known J-coupled multiplets within that same bin (e.g., doublets, triplets, etc.). Data were then autoscaled using various datasets as reference.

### 2.14. Metabolomics Profiling and Identification

For metabolomics profiling and identification, we conducted multivariate data analysis on binned scales spectral data using SIMCA-P+13 (Umetrics AB, Umeå, Sweden). The principal component analysis (PCA) was initially applied to analyze the NMR spectral data to separate different IF groups from non-IF samples. The data were visualized using the principal component (PC) score plots to identify general trends and outliers. PCA model constructs were based on specific experimental groups to explore any systematic differences between groups that may exist. Once the model is constructed, other groups can then be superimposed into the visualization by applying the model-specific bin coefficients (PCA loadings) to show how they compare. Partial least squares discriminant analysis (PLS-DA) was subsequently used to determine whether the fasting status, either regular or irregular, and non-fasting groups were different, as well as to identify the experimental variables that control the difference. To identify the variables contributed to the assignment of spectra between the experimental group and normal controls, we analyzed the variable importance in the projection (VIP) values of all peaks from PLS-DA model, and variables with VIP > 0.7 were considered relevant for group discrimination. Unpaired Student’s *t*-test (*p* < 0.05) to the chemical shifts was also used to assess the significance of each metabolite. Only both VIP >0.7 of multivariate and *p* < 0.05 of univariate statistical significance were identified as distinguishing metabolites. The corresponding chemical shift and multiplicity of the metabolites were identified by comparisons with the previous literature and the Human Metabolome Database (http://www.hmdb.ca/), a web-based bioinformatics/cheminformatics resource with detailed information about metabolites and metabolic enzymes. Then, the MetaboAnalyst version 4.0 software was used to perform the pathway analysis to identify the possible metabolite pathways that were associated with IF. 

### 2.15. Statistical Analysis

Statistical analysis was conducted using SPSS version 22 (IBM Corp., Armonk, NY, USA). Socio-demographic data were compared among the three IF groups by using the one-way ANOVA and chi-square test. Anthropometry data, biochemical profile, cognitive performance, level of oxidative stress, DNA damage, and level of the inflammatory marker were compared across IF status at baseline and follow-up after 36 months using the repeated measures ANCOVA with adjustments of covariates such as age and education year. This statistical analysis was used to determine the time–treatment interaction effects of within–between group effects. For within–between group analyses, the results were interpreted by the *p*-value of the F-test and the estimated marginal means. The mean for one group did not overlap with the corresponding confidence interval of another group. All the assumptions for repeated measured covariance were met (normality of residuals and homogeneity of variance). *p*-values less than 0.05 were considered statistically significant.

## 3. Results

### 3.1. Socio-Demographic Data

As shown in Table 1, subjects who regularly practiced IF (*p* < 0.05) were married, non-smokers, and had a higher number of years of education. It was found that 78.4% of study subjects in the r-IF group were married while 21.6% of the group were categorized as widowers, separated, or single. The study also found that 88.9% of subjects who do not practice IF (n-IF) had less than 6 years of education. In addition, the n-IF group showed a significantly lower number of years of education when compared to the r-IF group (*p* < 0.05). Moreover, 56.8% of subjects from the r-IF group were found to be non-smokers, while 27% of them were ex-smokers. However, 55.6% of the subjects from the n-IF group were smokers, as compared to 16.2% from the r-IF group.

### 3.2. Anthropometry Analysis and Blood Pressure

There was significant difference in mean weight, body mass index (BMI), waist circumference, and hip circumference among three different IF groups across time (*p* < 0.05). The mean weight in r-IF (52.84 ± 1.47 kg) at baseline decreased significantly after 36 months (49.19 ± 1.48 kg), while the n-IF group showed significant increment in mean weight across time (63.99 ± 1.72 kg vs. 66.27 ±1.73 kg). Moreover, the r-IF group also showed a significant drop in BMI and waist circumference at 36 months follow-up, as shown in Table 2. Overall, the weight, BMI, waist circumference, and hip circumference of the r-IF group was significantly (*p* < 0.05) lower than the n-IF group.

On the other hand, there was significant difference in systolic and diastolic blood pressure among three different IF groups across time (*p* < 0.001). The mean systolic and diastolic blood pressure of r-IF group at 36 months follow-up (132.31 ± 2.49 mmHg; 72.97 ± 1.70 mmHg) was significantly lower when compared to baseline (139.35 ± 2.83 mmHg; 76.76 ± 1.75 mmHg). The mean systolic and diastolic blood pressure of the r-IF group was also significantly lower than the n-IF group at 36 months follow-up, as shown in Table 2. 

### 3.3. Biochemical Profiles

There were significant differences in mean fasting blood sugar levels (*p* < 0.01), high-density lipoprotein (HDL) levels (*p* < 0.001), triglyceride levels (*p* < 0.05), total cholesterol levels (*p* < 0.05), and insulin levels (*p* < 0.001) among three different IF groups across time (Table 2). The r-IF group had significantly lower mean fasting blood glucose levels and higher HDL levels at 36 months follow-up as compared to baseline. On the other hand, the mean fasting blood glucose levels and triglyceride levels in the n-IF group increased significantly after 36 months while the HDL levels decreased significantly after 36 months. The i-IF group had significantly higher HDL levels and lower LDL, triglyceride, and total cholesterol levels at 36 months follow-up as compared to baseline. At 36 months follow-up, the LDL, triglyceride, and total cholesterol of the r-IF group were significantly lower than the i-IF and n-IF groups. Meanwhile, the mean insulin levels of the r-IF group decreased significantly after 36 months (92.97 ± 0.88 pmol/L) as compared to the baseline (112.61 ± 0.96 pmol/L). However, the mean insulin levels of i-IF (129.00 ± 0.98 pmol/L vs. 131.97 ± 0.91 pmol/L) and n-IF groups (140.83 ± 0.24 pmol/L vs. 129 ± 0.98 vs. 168.14 ± 0.31 pmol/L) showed increment across time. Generally, the insulin levels of the r-IF group were significantly lower when compared to the i-IF and n-IF groups at 36 months follow-up.

### 3.4. Analysis of DNA Damage

**Figure 1:**
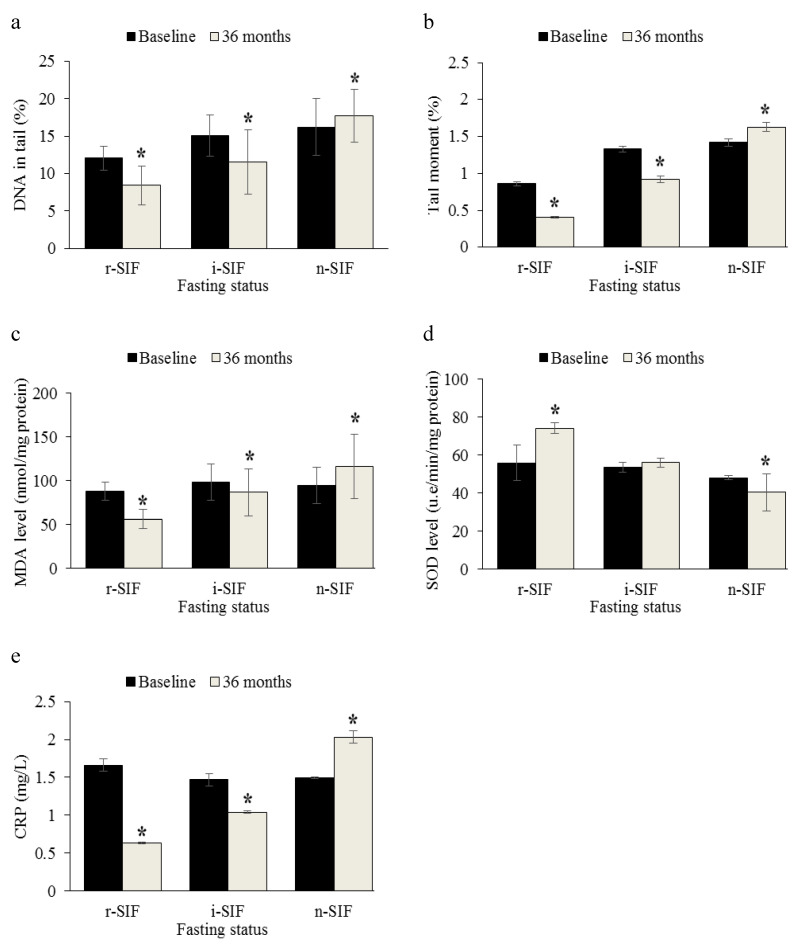
Effects of intermittent fasting (IF) on DNA damage, oxidative stress and inflammatory markers in mild cognitive impairment-afflicted older adults. (**a**) Percentage of DNA in tail, (**b**) percentage of tail moment, (**c**) malondialdehyde (MDA) levels, (**d**) superoxide dismutase (SOD) activities, and (**e**) C-reactive protein (CRP) levels among three different IF groups across time. Repeated measures analysis of covariance (ANCOVA) between group analyses with regard to time was applied. Numerical covariate (age, level of education) was controlled using repeated measures ANCOVA. * Significant difference as compared to baseline (*p* < 0.05). CRP: C-reactive protein; MDA: malondialdehyde; SOD: superoxide dismutase.

Figure 1a,b shows that there were significant differences in the mean percentage of DNA in tail and mean percentage of tail moment among three different IF groups across time (*p* < 0.001). Mean percentage of DNA in tail in r-IF (12.06 ± 1.61%) and i-IF groups (15.09 ± 2.79%) at baseline decreased significantly after 36 months (8.44 ± 2.59% and 11.58 ± 4.27%, respectively), whilst the percentage of DNA in tail at baseline (16.24 ± 3.76%) for n-IF groups increased significantly after 36 months (17.73 ± 3.53%). The data also showed that the mean percentage of tail moment increased significantly in the n-IF group (1.42 ± 0.05% vs. 1.63 ± 0.06%) across time as compared to the percentage of tail moment in r-IF (0.86 ± 0.03% vs. 0.41 ± 0.01%) and i-IF groups (1.33 ± 0.04% vs. 0.92 ± 0.05%).

### 3.5. Analysis of Oxidative Stress Markers

Figure 1c,d shows that there were significant differences in mean MDA levels and SOD activities among three different IF groups across time (*p* < 0.001). The data also showed that mean MDA decreased significantly in r-IF (87.58 ± 10.23 nmol/mg protein vs. 56.10 ± 10.41 nmol/mg protein) and i-IF groups (92.98 ± 4.75 nmol/mg protein vs. 86.33 ± 7.31 nmol/mg protein) after 36 months, while the mean MDA in the n-IF group showed increment across time (94.34 ± 5.33 nmol/mg protein vs. 116.01 ± 6.82 nmol/mg protein). On the other hand, the mean SOD activity decreased significantly in the n-IF group (47.92 ± 1.04 u.e/min/mg protein vs. 40.39 ± 9.84 u.e/min/mg protein), while the SOD activity in r-IF (55.73 ± 9.31 u.e/min/mg protein vs. 74.11± 2.89 u.e/min/mg protein) showed increment across time. There were no significant changes in SOD activity across time in the i-IF group (53.60 ± 2.54 u.e/min/mg protein vs. 55.91 ± 2.38 u.e/min/mg protein).

### 3.6. Analysis of Inflammatory Marker

Figure 1e shows that there were significant differences in mean CRP levels among the three different IF groups across time (*p* < 0.001). The data also showed that mean CRP levels decreased significantly in r-IF (1.66 ± 0.08 nmol/mg protein vs. 0.63 ± 0.01 nmol/mg protein) and i-IF groups (1.47 ± 0.08 nmol/mg protein vs. 1.04 ± 0.02 nmol/mg protein), while the mean CRP levels in n-IF group (1.49 ± 0.01 nmol/mg protein vs. 2.03 ± 0.08 nmol/mg protein) showed significant increment across time. Our current findings showed that subjects who did not practice IF regularly had a higher level of inflammatory markers.

### 3.7. Cognitive Performance

There were significant differences in mean scores for Digit Span (*p* < 0.001), RAVLT (*p* < 0.001), MMSE (*p* < 0.001), MoCA (*p* < 0.001), and Digit Symbol (*p* < 0.05) among the three different IF groups across time (Table 2). The data showed that mean scale score for Digit Span was significantly higher in r-IF (7.84 ± 1.86 vs. 8.88 ± 2.38) and i-IF groups (6.76 ± 2.49 vs. 7.32 ± 2.21) as compared to mean scale score for Digit Span in the n-IF group (7.76 ± 2.28 vs. 5.73 ± 2.44) across time. Meanwhile, T-score for RAVLT was significantly lower in the n-IF group (24.70 ± 0.77 vs. 20.41 ± 0.71) as compared to T-score for RAVLT in r-IF (30.00 ± 0.66 vs. 36.05 ± 0.61) and i-IF groups (30.21 ± 0.68 vs. 32.14 ± 0.62), which showed significant increment across time.

Mean MMSE score was significantly higher in the r-IF group (19.59 ± 2.05 vs. 24.05 ± 3.25) and i-IF group (18.60 ± 2.38 vs. 22.40 ± 3.38) as compared to mean MMSE score in the n-IF group across time (18.73 ± 1.48 vs. 16.33 ± 4.11). For MoCA, it was shown that the r-IF group (16.82 ± 4.10 vs. 19.43 ± 4.45) and i-IF groups (15.84 ± 2.73 vs. 19.00 ± 2.04) had significantly higher scores as compared to the n-IF group across time. Mean scale score for Digit Symbol decreased significantly in the n-IF group (4.43 ± 1.33 vs. 3.33 ± 1.81) compared to other IF groups across time.

Table 3 shows that at 36 months follow-up, subjects with MCI who regularly practiced IF showed changes in their cognitive function status. Out of 37 subjects who regularly practiced IF, 24.3% of the subjects were categorized as successful aging after 36 months. Meanwhile, 73.0% of subjects reverted into normal aging and the remaining 2.7% of subjects maintained MCI. Results from this present study showed that regularly practicing IF could help to improve cognitive function.

### 3.8. Metabolite Identification

To determine the differences in the metabolite profile of the r-IF, i-IF, and n-IF groups, we used PCA to analyze the ^1^H NMR data after normalization. The results showed an apparent separation among the three IF groups on the scores plot of PCA 1 (t [1]) (Figure S1). The majority of the samples were located in a 95% confidence interval. 

To identify the main metabolites that were responsible for the separation between the r-IF, i-IF, and n-IF groups, we obtained their scores and loadings plots with correlation coefficients from PLS-DA analysis on the basis of the NMR data. The scores plot of PLS 1 (t [1]) showed that the r-IF and i-IF groups were clearly distinguishable from n-IF group. The loading columns showed the significant class-discriminating metabolites responsible for the clustering patterns.

This study identified that most of the metabolites present in r-IF groups were associated with cognitive function improvement. Our study showed that a panel of 21 metabolites in plasma with VIP > 0.7 and *p* < 0.05 from the Student’s *t*-test were identified, as summarized in Table 4. Then, the representative metabolites with significant differences in plasma samples were represented in box-and-whisker plots (Figure S2), which showed the concentration ranges, median quartiles, and extremes. The data showed that isoleucine, 3-hydroxybutyrate, 3-hydroxy-3-methylglutaryl coenzyme A (CoA), glutathione, acetoacetate, n-acetylglucosamine, guanosine, and glucose were metabolites that were significantly present in the plasma of the r-IF and i-IF groups. On the other hand, valine, alanine, 4-hydroxybutyrate, s-sulfocysteine, 2′-deoxyguanosine, and phenylalanine were significantly present in n-IF groups. The data also showed that the concentration of glutamate, alanine, 4-hydroxybutyrate, s-sulfocysteine, and 2-deoxyguanosine were higher in the n-IF group as compared to the other IF groups.

Pathway analysis showed that synthesis and degradation of ketone bodies, glutathione metabolism, alanine, aspartate and glutamate metabolism, phenylalanine metabolism, arginine and proline metabolism, and D-glutamine and D-glutamate metabolism pathways had an impact value of >0.1 with a *p*-value of <0.05 (Table 5). Thus, our current findings suggested that alteration in such metabolite pathways may affect the cognitive performance in older adults with different fasting statuses. Although the impact value of other pathways such as butanoate metabolism; valine, leucine, and isoleucine degradation; propanoate metabolism; metabolism of taurine and hypotaurine; valine, leucine, and isoleucine biosynthesis; terpenoid backbone biosynthesis; starch and sucrose metabolism; aminoacyl-transfer RNA (tRNA) biosynthesis; selenoamino acid metabolism; glycolysis or gluconeogenesis; and nitrogen metabolism did not exceed 0.1, they were still significant in terms of *p*-value.

## 4. Discussion

In this study, socio-demographic data showed that older adults who practiced IF regularly received better education compared to the other groups. It is possible that receiving a higher level of education may have created awareness that IF has a beneficial effect on health and cognitive function. Moreover, most of the subjects in the r-IF group were married. Previously, it has been reported that social or family support can increase the successfulness in the continuation of IF practice [23]. Furthermore, subjects who regularly practiced IF were mostly non-smokers. This showed that fasting or changes in dietary habit could reduce the addiction towards tobacco. Furthermore, tobacco smoke itself also influences food choice. Nicotine has been postulated to influence consumer perception on smell and taste of food, and hence leading smokers to prefer meat and fat-rich foods as an alternative to fruits and vegetables. In relation to diet changes, a previous study has shown that after smoking cessation, diet changes becomes more similar to that of non-smokers [24]. Self-controlled fasting also aids in reducing smoking desires. Another study has shown that subjects that consumed a low energy diet experienced less cravings toward cigarettes and increased appetite. These results are quite similar to this current study, where IF could reduce the desire for cigarettes [25].

The r-IF group demonstrated an overall improvement in weight status, BMI, and waist circumference at 36 months follow-up. Our current findings are in agreement with a previous study that reported that participants who practiced intermittent fasting for 24 months showed a 3–4% reduction in body weight [26]. Meanwhile, the n-IF group showed an increment in body weight, BMI, waist circumference, and hip circumference at 36 months follow-up. This was probably due to lack of dietary control in the daily routine. It is noted that the BMI value of n-IF group also exceeded the normal BMI range, indicating that the subjects in the n-IF group were more likely to have overweight/obesity-related diseases. In fact, most of the subjects in this group had problems with hypertension, diabetes, and hypercholesterol. Excess weight and obesity are also associated with decreased cognitive function, as well as dementia [27,28]. High BMI value may adversely affect the structure and function of the brain, such as reduction in prefrontal metabolism and temporal lobe atrophy [29,30]. Moreover, as compared to overweight and normal individuals, obese subjects showed a reduction in overall brain volume and gray matter as well [31].

Subjects who practiced IF regularly also showed improvement in the blood pressure readings at 36 months follow-up. Weight and obesity are known to be independent risk factors for high blood pressure [32]. In this present study, we also found out that decreased in blood pressure was parallel to weight loss in the r-IF group. Furthermore, other fasting and CR studies generally showed beneficial results on blood pressure due to the reduction in sodium intake during fasting and consuming low-calorie diets [33,34]. Moreover, previous studies demonstrated that blood pressure was associated with impairment in cognitive function [35,36]. This is in line with our current findings, since subjects with high blood pressure also exhibited deterioration in cognitive function. High blood pressure can trigger blood–brain barrier dysfunction, which may lead to the penetration of proteins from the blood into the brain tissue. The proteins that enter into the brain can interact with neurons or synapses as well as inducing the accumulation of amyloid-β in the brain [37]. Thus, our present results suggest that practicing IF can help to improve cognitive function by reducing blood pressure, possibly due to weight loss.

The present study demonstrated that MDA levels were significantly lower among the older adults who practice IF regularly, suggesting that IF may help to reduce the level of oxidative stress and protect against lipid peroxidation in the body. In agreement with the previous study, this study showed that the reduction in MDA levels were accompanied with significant reduction in body weight in r-IF groups [38,39]. This weight loss could be attributed to the decrease in fat mass percentage and/or the decrease in fluid intake and partial dehydration [40]. Decrease in fat mass has been suggested to lower production of free radical from fat accumulation, leading to reduced level of oxidative stress. Moreover, our current findings showed that the SOD activity was significantly higher among older adults that regularly practiced IF, implying that IF could aid in reducing the oxidative stress by increasing antioxidant defense mechanisms. Therefore, it is likely that reduction in oxidative stress could reduce the incidence of oxidative damage among older adults with MCI after 36 months of regularly practicing IF.

Furthermore, DNA damage can occur in response to oxidative stress [41]. Previous research findings have stated that DNA damage events increased with age due to the inability of the body to fight against the free radicals that are produced in cells [42,43]. If DNA damage is very severe or exceeds the capacity of DNA repair mechanisms, cell aging or apoptosis can occur, and this may contribute to the aging process and lead to cognitive dysfunction [42,44]. Our current results showed that subjects who practiced IF regularly had significant lower DNA damage events, possibly due to the increase in SOD activities, as observed in the r-IF group. Similarly, subjects who practiced CR for 6 months were shown to have a lower DNA damage level as compared to baseline [45]. Fasting was demonstrated to activate the Krebs cycle, which produces intermediate molecules that act as electron donors to antioxidant molecules [46]. This shows that the practicing of IF can increase the antioxidant defenses in the body and reduce DNA damage events. Moreover, CR was also found to increase the activity of some enzymes, including the DNA-repairing enzymes [47]. Therefore, IF is expected to maintain the cognitive function of older adults by reducing DNA damage events.

Previously, a study conducted on obese women found that the CRP levels decreased following 28 days of low-calorie diet intake [48]. It has been demonstrated that fasting can reduce systemic inflammation due to weight reduction after IF practice [49]. This is because inflammation occurs when the liver releases free fatty acids and triacylglycerol. It promotes the adipose tissue to produce cytokines (IL-6) and leads to the secretion of CRP [50]. Our current findings are in agreement with a previous study since the IF group was found to have normal BMI, lower triglycerides levels, and lower CRP concentrations during 36 months follow-up, indicating that the reduction in CRP levels was due to the reduction in body weight and fat accumulation.

Our current findings showed that practicing IF can reduce insulin levels following the 36 months of the study. A previous study showed that in obese individuals, insulin and fasting blood glucose levels decreased after 4 days of CR, hence improving the glucose tolerance and insulin sensitivity [51,52]. Furthermore, insulin resistance may also impair cognitive function [53]. This shows that suitable diet regime such as practicing IF may affect insulin sensitivity and reduce insulin resistance in older adults, hence improving their cognitive functions. Moreover, insulin has been shown to affect the growth and apoptosis in neurons [54]. The elevation of insulin level would disturb the biochemistry of the brain [55]. One of the possible mechanisms could be the modification of insulin signaling that leads to the formation of amyloid plaques, changes in amyloid-β metabolism, and deposition of tau hyperphosphorylation [56]. Spending more time in a fed state and less time in a fasted state cause insulin levels to elevate over the time and prevents the body from burning stored body fat. Hyperinsulinemia stimulates the release of intracellular amyloid-β and inhibits its degradation by insulin-degrading enzyme [57]. Deposition of amyloid-β in the brain is neurotoxic and causes acute memory-inhibiting effects. Thus, reduced insulin levels as observed in IF practitioners after 36 months of follow-up may help to reduce the major adverse metabolic events that can affect the cognitive functions in older adults with MCI. Moreover, insulin resistance due to high insulin level is also associated with an increased inflammatory condition, including elevated CRP [58]. Hence, the reduction in CRP levels among those who practice IF regularly might also be due to the reduction in insulin levels, as observed in this present study.

Cognitive tests used in this present study detected a wide range of psychological domains, focusing more on global function, including orientation, memory, registration, calculation, attention, and language. We found that IF significantly enhanced cognitive performance among older adults. MMSE, MoCA, RAVLT, Digit Symbol, and Digit Span scores were better in older adults who practiced IF. Interestingly, our current findings showed that regularly and consistently practicing IF can help in reverting older adults with MCI during baseline to better cognitive function during follow-up (successful aging or normal aging). Previously, it has been demonstrated that up to 30% of CR was able to improve the verbal memory among individuals aged 50–80 years old after 3 months of intervention, possibly due to the reduction in insulin and CRP levels [59]. Furthermore, obese older adults with MCI who practiced CR for 12 months showed improvement in verbal memory, verbal fluency, executive function, and global cognition [60]. Moreover, our results are similar to the previous findings whereby fasting in Ramadan had been shown to associate with significant changes in cognitive function in healthy subjects [61]. However, another study demonstrated that Ramadan fasting did not give significant effect on visual learning and working memory among the athletes [62]. These results suggested that the effect of fasting on cognitive function is heterogeneous and domain-specific. This discrepancy could be due to differences in subjects’ socio-demographic profile, in the sampling procedure, and in the cognitive test used.

Since metabolomics provide a favorable opportunity to generate novel biomarkers and hypotheses for addressing the molecular mechanism of diseases, this study investigated the metabolite profiles of plasma among older adults with MCI of different IF groups [63]. Results from this present study clearly indicated that the practice of IF was related to lipid metabolism (synthesis and degradation of ketone bodies and butanoate metabolism). During fasting, there is a noticeable reduction in blood glucose levels and a rise in ketone bodies production by the liver due to metabolic adaptation. Results from metabolomics analysis showed that the levels of 3-hydroxybutyrate, 3-hydroxy-3-methylglutaryl-CoA, and acetoacetate were significantly higher among subjects that regularly practice IF. This result is consistent with a previous study whereby the level of acetoacetate and 3-hydroxybutyrate were higher among fasted groups [64]. This is because during fasting, glycogen that is stored in the liver is depleted. Then, fatty acid originating from the adipose tissue is oxidized to acetyl-CoA in the liver and converted to 3-hydroxy-3-methylglutaryl-CoA, which eventually converts to acetoacetate and 3-hydroxybutyrate, whereas ketone bodies are released into the blood and transported into the brain. Hence, the elevation of ketone bodies is possibly due to fatty acid oxidation.

Our current findings showed that IF could help in improving cognitive function through ketogenesis action. The production of the ketone bodies positively impacted cognitive function because it acts as an energy substrate and increases the neuron survival under pathological conditions such as hypoxia, anoxia, or ischemia [38]. Besides this, ketone bodies have been shown to have neuroprotective properties by increasing ATP levels and reducing ROS production through enhanced nicotinamide adenine dinucleotide (NADH) oxidation and inhibition of mitochondrial permeability transition [65]. A previous study also demonstrated that ketones can block the entry of amyloid-β 42, which is the pathologic hallmark protein of Alzheimer’s disease (AD) into neurons. The suppression of intracellular amyloid-β 42 accumulations rescued mitochondrial complex I activity, reduced oxidative stress, and improved synaptic plasticity. Thus, this study suggested that IF possibly exerts neuroprotective effects by undergoing the ketogenic pathway, hence blocking the accumulation of amyloid-β 42 [66]. Moreover, IF is also believed to have a protective effect against neurofibrillary tangles and tau-protein aggregation. Mice fed a precursor of ketone bodies showed reduced amyloid-β and hyperphosphorylated tau deposition in the hippocampus, amygdala, and cortex [67]. These results reveal that long-term feeding of ketone bodies may potentially retard the disease process and improve cognitive function of patients with AD [68]. Hence, our current findings suggest that IF could benefit older adults with MCI in relation to their cognitive function since practicing IF has been shown to increase the production of ketone bodies. This is in parallel with our current findings, where the older adults who practice IF who had MCI at the baseline underwent a positive change in cognitive function, wherein 24.3% was reverted to successful aging after 36 months of follow-up.

Glucose metabolism (glycolysis and gluconeogenesis, as well as the metabolism of amino sugars and nucleotide sugars) was also found to play a major role in IF practice. Indeed, the glucose and n-acetylglucosamine levels were significantly higher in the IF practicing groups than in the n-IF group. Glucose is important in ensuring the sustainability of cognitive function. During fasting, hepatic gluconeogenesis is a major source of endogenous glucose production [69]. Therefore, in this study, glucose metabolites can also be identified in the IF practice group. This is due to the production of endogenous glucose by the liver to ensure the stability of glucose supply for use by the brain as a source of energy.

The isoleucine metabolites were found to be higher in the n-IF group. This may be due to uncontrolled and inconsistent food intake. Branched chain amino acids (BCAA) such as isoleucine and valine are usually obtained from a high-protein diet. Proline is also a metabolite obtained from high-protein food sources such as meat and fish. Increased in BCAA metabolites and proline can affect cognitive function. Previous studies have demonstrated that high concentrations of BCAA can increase oxidative stress and inflammation. Some studies have shown that BCAA are neurotoxic and high levels of BCAA can increase the excitotoxicity in cortical neuron cells [70]. Furthermore, the phenylalanine metabolites were also found to be higher in the n-IF group. Phenylalanine plays a major role in the biosynthesis of other amino acids. Although phenylalanine is crucial in the production of proteins or other molecules, the accumulation of phenylalanine can cause toxicity and damage to neuronal cells, which in turn disrupt the cognitive function [71]. High concentrations of phenylalanine in the cerebral cortex of mice have also been reported to induce the occurrence of oxidative stress and reduce the viability of astrocytes [72]. On the other hand, the glutathione metabolite pathway was found to be activated in the n-IF group. This was probably due to the occurrence of oxidative stress as a result of increase concentration of BCAA among subjects that did not practice IF. The glutathione metabolism pathway is activated in order to protect cells from oxidative damage since glutathione is one of the main cellular antioxidant defense mechanisms [73].

The glutamate levels were also found to be higher among subjects who do not practice IF. Glutamate can serve as a precursor in the biosynthesis of amino acids such as L-proline and L-arginine [74,75]. This indicates that in this study metabolites such as proline are synthesized in the presence of glutamate. Increase in glutamate concentration in the plasma of the n-IF group was probably due to overconsumption of foods high in glutamate. Glutamate acts as an important signaling molecule in neurons, and accumulation of glutamate in the synapse has been reported to kill neuronal cells due to extreme excitotoxicity [76]. Moreover, increase in the concentration of 2′-deoxyguanosine was observed in the n-IF group as well, probably due to the increment in purine metabolism. A previous community study demonstrated that high levels of 2′-deoxyguanosine are associated with low cognitive scores [77]. Furthermore, evidence from an animal study has also proven that a high level of 2′-deoxyguanosine can lead to neuronal death by inducing the occurrence of oxidative stress in neuron cells [78].

## 5. Conclusions

In conclusion, the MCI-afflicted older adults who practiced IF regularly had better cognitive scores and reverted to better cognitive function groups (successful aging and normal aging) at 36 months follow-up. This study revealed that IF can offer similar benefits as compared to CR and other fasting diets. Furthermore, our current findings also suggest that IF could be a better solution to combat the cognitive impairment that occurs during the aging process since IF is more applicable and easy to practice, especially among older adults and people under clinical intervention.

## Figures and Tables

**Table 1:** Socio-demographic data of subjects from each group at 36 months follow-up (*n* = 99).

Characteristics		r-IF (*n* = 37) Number (%) or Mean ± SD	i-IF (*n* = 35) Number (%) or Mean ± SD	n-IF (*n* = 27) Number (%) or Mean ± SD	*p*-Value
Age (mean)		68.7 ± 4.6	67.9 ± 5.6	69.1 ± 5.0	0.65
Gender	Male	23 (62.2)	15 (42.9)	15 (55.6)	0.25
	Female	14 (37.8)	20 (57.1)	12 (44.4)	
Marital status	Married	29 (78.4)	22 (62.9)	11 (40.7)	0.01 *
	Single/divorced/widowed	8 (21.6)	13 (37.1)	16 (59.3)	
Years of education (year)	≤6 years	24 (64.9)	30 (85.7)	24 (88.9)	0.03 *
	>6 years	13 (35.1)	5 (14.3)	3 (11.1)	
Living status	Alone	1 (2.7)	4 (11.4)	6 (22.2)	0.05
	With others	36 (97.3)	31 (88.6)	21 (77.8)	
Smoking habits	Active smoker	6 (16.2)	15 (42.9)	15 (55.6)	0.01 *
	Ex-smoker	10 (27.0)	5 (14.2)	1 (3.7)	
	Non-smoker	21 (56.8)	15 (42.9)	11 (40.7)	

* Significant differences (*p* < 0.05) among groups.

**Table 2:** Comparison of anthropometry profiles, blood pressure, biochemical profiles, and cognitive test performance among three different intermittent fasting (IF) status groups on the basis of time (time*treatment interaction effect).

Parameter	Fasting Status	Baseline		36 Months		*p*-Value (Partial Eta Square)		
		Mean ± SD	95% CI	Mean ± SD	95% CI	Effect of Time	Effect of Group	Interaction Effect
Anthropometry								
Weight (kg)	r-IF (*n* = 37)	52.84 ± 1.47	49.92, 55.75	49.19 ± 1.48 ^###,a,b^	46.26, 52.12	0.12 (0.01)	<0.001 (0.34) *	0.03 (0.47) *
	i-IF (*n* = 35)	61.47 ± 1.51	58.47, 64.47	61.11 ± 1.52 ^a^	58.09, 64.12			
	n-IF (*n* = 27)	63.99 ± 1.72	60.57, 67.40	66.27 ± 1.73 ^###,b^	62.84, 69.71			
BMI (kg/m^2^)	r-IF (*n* = 37)	24.17 ± 0.53	23.11, 25.24	22.64 ± 0.52 ^###,a,b^	21.60, 23.68	0.02 (0.09) *	<0.001 (0.59) *	<0.001 (0.31) *
	i-IF (*n* = 35)	26.14 ± 0.50	25.15, 27.14	27.35 ± 0.48 ^##,a,c^	26.39, 28.33			
	n-IF (*n* = 27)	30.03 ± 0.63	28.78, 31.27	33.56 ± 0.61 ^###,b,c^	32.34, 34.78			
WC (cm)	r-IF (*n* = 37)	86.63 ± 1.32	84.02, 89.24	81.53 ± 1.57 ^###,b^	78.42, 84.65	0.02 (0.06) *	<0.001 (0.93) *	0.03 (0.04) *
	i-IF (*n* = 35)	87.46 ± 1.35	84.78, 90.15	87.81 ± 1.61 ^c^	83.61, 90.02			
	n-IF (*n* = 27)	92.85 ± 1.54	89.79, 95.91	95.80 ± 1.84 ^###,b,c^	92.16, 99.45			
HC (cm)	r-IF (*n* = 37)	90.59 ± 1.09	88.43, 92.75	90.22 ± 0.97 ^b^	88.29, 92.15	<0.001 (0.12) *	0.06 (0.05)	<0.001 (0.17) *
	i-IF (*n* = 35)	90.87 ± 1.12	88.65, 93.09	92.47 ± 1.29 ^#^	89.17, 94.33			
	n-IF (*n* = 27)	91.75 ± 1.29	89.17, 94.33	96.35 ± 1.16 ^###,b^	94.05, 98.65			
Blood pressure								
Systolic (mmHg)	r-IF (*n* = 37)	139.35 ± 2.83	133.74, 144.96	132.31 ± 2.49 ^###,a,b^	127.36, 137.27	0.74 (0.001)	0.01 (0.09) *	<0.001 (0.21) *
	i-IF (*n* = 35)	142.65 ± 2.91	136.89, 148.43	145.46 ± 2.57 ^a^	140.36, 150.55			
	n-IF (*n* = 27)	143.48 ± 2.57	136.91, 150.05	151.17 ± 2.92 ^##,b^	145.37, 156.97			
Diastolic (mmHg)	r-IF (*n* = 37)	76.76 ± 1.75	73.29, 80.22	72.97 ± 1.70 ^##,b^	69.59, 76.35	0.38 (0.01)	0.13 (0.04)	0.02 (0.08) *
	i-IF (*n* = 35)	78.06 ± 1.79	74.49, 81.62	76.98 ± 1.75	73.52, 80.46			
	n-IF (*n* = 27)	77.93 ± 2.05	73.87, 81.99	81.24 ± 1.99 ^#,b^	77.29, 85.19			
Biochemical profile								
FBS (mmol/L)	r-IF (*n* = 37)	5.49 ± 0.72	5.21,5.77	5.12 ± 0.60 ^##^	4.63, 5.62	0.15 (0.02)	<0.001 (0.17) *	0.004 (0.11) *
	i-IF (*n* = 35)	5.97 ± 1.11	5.61, 6.34	6.12 ± 1.43 ^c^	5.56, 6.67			
	n-IF (*n* = 27)	5.76 ± 0.94	5.44, 6.09	6.92 ± 2.40 ^#,c^	6.64, 7.56			
HDL (mmol/L)	r-IF (*n* = 37)	1.42 ± 0.27	1.33, 1.52	1.75 ± 0.39 ^###,a^	1.65, 1.86	0.008 (0.07) *	<0.001 (0.27) *	<0.001 (0.24) *
	i-IF (*n* = 35)	1.25 ± 0.37	1.13, 1.38	1.39 ± 0.39 ^###,a^	1.25, 1.54			
	n-IF (*n* = 27)	1.24 ± 0.31	1.12, 1.35	1.05 ± 0.29 ^#^	0.92, 1.18			
LDL (mmol/L)	r-IF (*n* = 37)	3.20 ± 0.66	2.98, 3.43	2.77 ± 0.61 ^a,b^	2.51, 3.03	0.003 (0.08) *	0.046 (0.06) *	0.05 (0.06)
	i-IF (*n* = 35)	3.56 ± 0.74	3.26, 3.86	3.07 ± 0.84 ^###,a^	2.73, 3.41			
	n-IF (*n* = 27)	3.56 ± 0.89	3.29, 3.84	3.77 ± 1.13 ^b^	3.44, 4.07			
TG (mmol/L)	r-IF (*n* = 37)	1.66 ± 0.51	1.43, 1.89	1.46 ± 0.68 ^a,b^	1.13, 1.76	0.604 (0.03)	<0.001 (0.18) *	0.02 (0.08) *
	i-IF (*n* = 35)	2.14 ± 0.98	1.84, 2.45	1.65 ± 0.84 ^###,a,c^	1.23, 2.06			
	n-IF (*n* = 27)	2.39 ± 0.88	2.11, 2.67	2.94 ± 1.51 ^###,b,c^	2.57, 3.32			
TC (mmol/L)	r-IF (*n* = 37)	4.03 ± 0.82	3.73, 4.34	3.57 ± 1.09 ^a,b^	3.22, 3.93	0.002 (0.10) *	0.06 (0.06)	0.02 (0.08) *
	i-IF (*n* = 35)	4.41 ± 1.33	3.99, 4.79	3.68 ± 1.29 ^##,a^	3.21, 4.14			
	n-IF (*n* = 27)	4.30 ± 0.97	3.94, 4.68	4.53 ± 1.15 ^b^	4.11, 4.96			
INS (pmol/L)	r-IF (*n* = 37)	112.61 ± 0.96	110.70, 114.52	92.97 ± 0.88 ^###,a,b^	91.23, 94.72	0.002 (0.10) *	<0.001 (0.94) *	<0.001 (0.76) *
	i-IF (*n* = 35)	129.00 ± 0.98	127.39, 131.32	131.97 ± 0.91 ^#,a,c^	130.17, 133.77			
	n-IF (*n* = 27)	140.83 ± 0.24	138.59, 143.06	168.14 ± 0.31 ^###,b,c^	146.09, 170.18			
Cognitive test performance								
Digit Span (scale score)	r-IF (*n* = 37)	7.84 ± 1.86	7.19, 8.48	8.88 ± 2.38 ^##,a,b^	8.18, 9.59	<0.001 (0.07) *	0.004 (0.13) *	<0.001 (0.19) *
	i-IF (*n* = 35)	6.76 ± 2.49	5.94, 7.65	7.32 ± 2.21 ^##,a^	7.42, 9.29			
	n-IF (*n* = 27)	7.76 ± 2.28	6.96, 8.52	5.73 ± 2.44 ^###,b^	4.86, 6.56			
RAVLT (T5 score)	r-IF (*n* = 37)	30.00 ± 0.66	29.23, 31.91	36.05 ± 0.61 ^###,b^	38.85, 37.25	<0.001 (0.28) *	<0.001 (0.62) *	<0.001 (0.66) *
	i-IF (*n* = 35)	30.21 ± 0.68	28.40, 32.02	32.14 ± 0.62 ^###,c^	30.91, 33.37			
	n-IF (*n* = 27)	24.70 ± 0.77	23.16, 26.24	20.41 ± 0.71 ^###,b,c^	19.00, 21.81			
MMSE (total score)	r-IF (*n* = 37)	19.59 ± 2.05	18.99, 20.18	24.05 ± 3.25 ^###,a,b^	22.98, 25.11	<0.001 (0.16) *	<0.001 (0.32) *	<0.001 (0.29) *
	i-IF (*n* = 35)	18.60 ± 2.38	17.84, 19.41	22.40 ± 3.38 ^###,a,c^	20.95, 23.78			
	n-IF (*n* = 27)	18.73 ± 1.48	17.99, 19.43	16.33 ± 4.11 ^##,b,c^	15.06, 17.65			
MoCA (total score)	r-IF (*n* = 37)	16.82 ± 4.10	15.70, 17.94	19.43 ± 4.45 ^###,b^	18.26, 20.61	<0.001 (0.18) *	0.002 (0.12) *	0.003 (0.11) *
	i-IF (*n* = 35)	15.84 ± 2.73	14.34, 17.32	19.00 ± 2.04 ^###,c^	17.42, 20.55			
	n-IF (*n* = 27)	14.77 ± 3.82	13.42, 16.13	14.37 ± 4.25 ^##,b,c^	12.95, 15.81			
Digit Symbol (scale score)	r-IF (*n* = 37)	4.11 ± 1.33	3.69, 4.53	5.39 ± 2.78 ^##,b^	4.70, 6.07	0.04 (0.64) *	0.19 (0.03)	<0.001 (0.16) *
	i-IF (*n* = 35)	3.64 ± 1.55	3.09, 4.20	4.68 ± 1.73 ^#^	3.76, 5.57			
	n-IF (n = 27)	4.43 ± 1.33	3.92, 4.94	3.33 ± 1.81 ^###,b^	2.52, 4.17			

Repeated measures analysis of covariance (ANCOVA) between group analyses with regard to time was applied. Numerical covariate (age, level of education) was controlled using repeated measures ANCOVA. Assumptions of normality, homogeneity of variances, compound symmetry, and homogeneity of regression were checked and were fulfilled. * *p* < 0.05; ^#^ significant difference (*p* < 0.05) as compared to baseline; ^##^ significant difference (*p* < 0.01) as compared to baseline; ^###^ significant difference (*p* < 0.001) as compared to baseline; ^a^ significant difference between regularly practicing IF (r-IF) and irregularly practicing IF (i-IF); ^b^ significant difference between r-IF and non-fasters (n-IF); ^c^ significant difference between i-IF and n-IF. BMI: body mass index; FBS: fasting blood sugar; HC: hip circumference; HDL: high-density lipoprotein; INS: insulin; LDL: low-density lipoprotein; MMSE: Mini Mental State Examination; MoCA: Montreal Cognitive Assessment; RAVLT: Rey Auditory Verbal Learning Test; TC: total cholesterol; TG: triglycerides; WC: waist circumference.

**Table 3:** Cognitive changes after 36 months follow-up (*n* = 99).

Cognitive Group (36 Months of Follow-Up)	Fasting Status Group			*p*-Value
	r-IF (*n* = 37)	i-IF (*n* = 35)	*n*-IF (*n* = 27)	
	Number (%)	Number (%)	Number (%)	
Successful aging (SA)	9 (24.3)	5 (14.2)	1 (3.7)	<0.001 *
Normal aging (UA)	27 (73.0)	22 (62.9)	8 (29.6)	
MCI	1 (2.7)	8 (22.9)	18 (66.7)	

* Significant differences (*p* < 0.05) among groups. MCI: mild cognitive impairment.

**Table 4:** Changes in plasma metabolites of mild cognitive impairment (MCI)-afflicted older adults in relation to fasting status.

Metabolite	δ^1^H (ppm)	Changes		
		r-IF vs. i-IF	r-IF vs. n-IF	n-IF vs. i-IF
Valine	1.04 (d)	x	x	+ *
3-Hydroxybutyrate	1.18 (m)	+ *	x	x
Isoleucine	1.24 (s)	─ *	+ *	x
3-Hydroxy-3-methylglutaryl-CoA	1.28 (s)	x	+ *	x
Alanine	1.48 (d)	x	─	+ *
Acetate	1.88 (s)	+ *	x	x
2-Aminoadipate	1.96 (d)	x	+ *	x
Glutamate	2 (m)	x	─ *	+ *
Glutathione	2.2 (s)	x	+ *	x
4-Hydroxybutyrate	2.28 (s)	x	─	+ *
Acetoacetate	3.42 (m)	+ *	x	x
S-sulfocysteine	3.48 (m)	x	─ *	+ *
n-Acetylglucosamine	3.68 (q)	+ *	─ *	─ *
2′-Deoxyguanosine	3.72 (m)	x	─ *	+ *
2-Hydroxybutyrate	3.92 (d)	x	x	+ *
Hippurate	3.96 (s)	x	─ *	+ *
Proline	4.16 (q)	x	x	+ *
Guanosine	4.44 (s)	+ *	─ *	x
Glucose	5.24 (d)	+ *	─ *	x
*n*-Acetylserotonin	7.04 (s)	+ *	x	x
Phenylalanine	7.32 (d)	x	x	+ *

Variable importance in the projection was obtained from the partial least squares discriminant analysis (PLS-DA) model with a threshold of 1.0. s: singlet; d: doublet; q: doublet of doublets; m: multiplet; +: a relatively higher concentration; −: a relatively lower concentration, x: cannot be detected. * *p* < 0.05.

**Table 5:** Potential metabolite pathway analysis by using MetaboAnalyst software.

Metabolite Pathway	*p*-Value	Holm Adjust	False Discovery Rate	Impact
Synthesis and degradation of ketone bodies	1.13 × 10^−5^	9.01 × 10^−4^	6.57 × 10^−4^	0.9
Glutathione metabolism	4.22 × 10^−2^	1.00	2.72 × 10^−1^	0.25
Alanine, aspartate, and glutamate metabolism	1.78 × 10^−2^	1.00	1.58 × 10^−1^	0.23
Phenylalanine metabolism	5.23 × 10^−2^	1.00	3.28 × 10^−1^	0.15
Arginine and proline metabolism	1.44 × 10^−2^	1.00	6.89 × 10^−1^	0.14
D-glutamine and D-glutamate metabolism	5.21 × 10^−2^	1.00	4.91 × 10^−1^	0.11
Pyruvate metabolism	2.46 × 10^−2^	1.00	8.08 × 10^−1^	0.1
Butanoate metabolism	1.64 × 10^−5^	1.30 × 10^−3^	6.57 × 10^−4^	0.09
Lysine biosynthesis	2.46 × 10^−1^	1.00	8.08 × 10^−1^	0.07
Aminoacyl-tRNA biosynthesis	2.80 × 10^−5^	2.19 × 10^−3^	7.47 × 10^−4^	0.06
Valine, leucine, and isoleucine degradation	3.19 × 10^−4^	2.46 × 10^−2^	6.39 × 10^−3^	0.06
Propanoate metabolism	3.13 × 10^−3^	2.38 × 10^−1^	5.01 × 10^−2^	0.03
Taurine and hypotaurine metabolism	1.25 × 10^−2^	9.27 × 10^−1^	1.43 × 10^−1^	0.03
Valine, leucine, and isoleucine biosynthesis	2.23 × 10^−2^	1.00	1.79 × 10^−1^	0.03
Sulfur metabolism	1.46 × 10^−1^	1.00	6.89 × 10^−1^	0.03
Terpenoid backbone biosynthesis	2.53 × 10^−1^	1.00	8.08 × 10^−1^	0.03
Purine metabolism	1.90 × 10^−1^	1.00	8.01 × 10^−1^	0.02
Lysine degradation	3.40 × 10^−1^	1.00	9.72 × 10^−1^	0.02
Starch and sucrose metabolism	3.58 × 10^−1^	1.00	9.87 × 10^−1^	0.02
Tryptophan metabolism	5.05 × 10^−1^	1.00	1.00	0.02
Cysteine and methionine metabolism	1.18 × 10^−2^	8.84 × 10^−1^	1.43 × 10^−1^	0.01
Amino sugar and nucleotide sugar metabolism	1.78 × 10^−1^	1.00	7.89 × 10^−1^	0.01
Selenoamino acid metabolism	1.51 × 10^−2^	1.00	1.51 × 10^−1^	0
Glycolysis or gluconeogenesis	2.90 × 10^−2^	1.00	2.11 × 10^−1^	0
Nitrogen metabolism	4.43 × 10^−2^	1.00	2.72 × 10^−1^	0
Pantothenate and CoA biosynthesis	2.12 × 10^−1^	1.00	8.07 × 10^−1^	0
Phenylalanine, tyrosine, and tryptophan biosynthesis	2.12 × 10^−1^	1.00	8.07 × 10^−1^	0
Pentose phosphate pathway	2.46 × 10^−1^	1.00	8.08 × 10^−1^	0
Galactose metabolism	3.04 × 10^−1^	1.00	9.35 × 10^−1^	0
Histidine metabolism	3.22 × 10^−1^	1.00	9.55 × 10^−1^	0
Tyrosine metabolism	4.92 × 10^−1^	1.00	1.00	0

## Supplementary Materials

The following are available online at https://www.mdpi.com/2072-6643/12/9/2644/s1, Figure S1: The PCA-X scatter plot, PLS-DA scatter plot, permutation test, and loading plot of the metabolomics analysis, Figure S2: Box-and-whisker plots for each plasma metabolite concentration in relation with fasting status.
