# Supplementary material for: Intermittent Fasting Enhanced the Cognitive Function in Older Adults with Mild Cognitive Impairment by Inducing Biochemical and Metabolic changes: A 3-Year Progressive Study
Source: Nutrients. 2020 Aug 30;12(9):2644. doi: 10.3390/nu12092644 (PMC7551340; doi:10.3390/nu12092644)
Supplement: Supplementary file 1 [file nutrients-12-02644-s001.pdf]

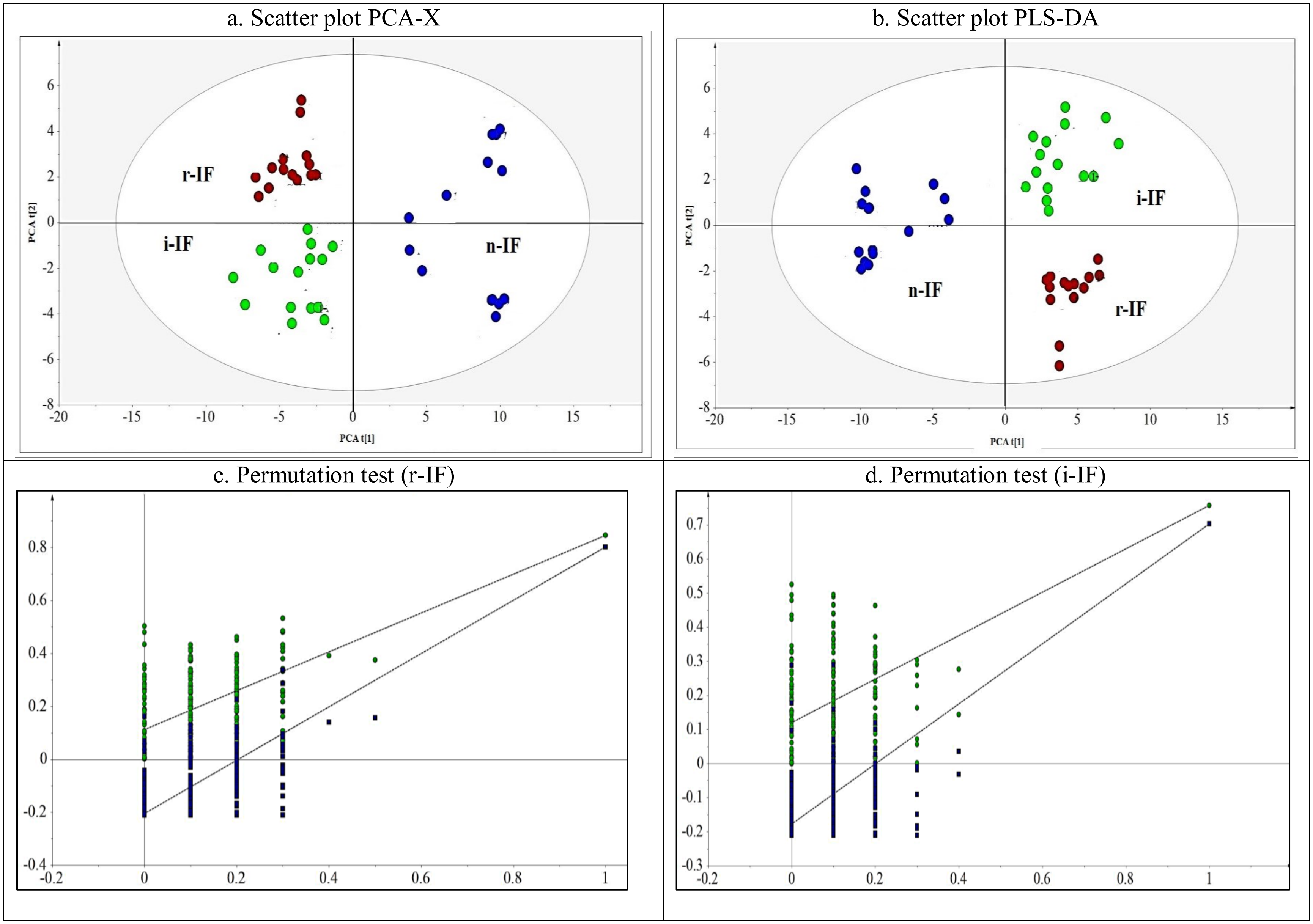
a. Scatter plot PCA-X
b. Scatter plot PLS-DA
c. Permutation test (r-IF)
d. Permutation test (i-IF)
r-IF
i-IF
n-IF
PCA t[1]
PCA t[2]

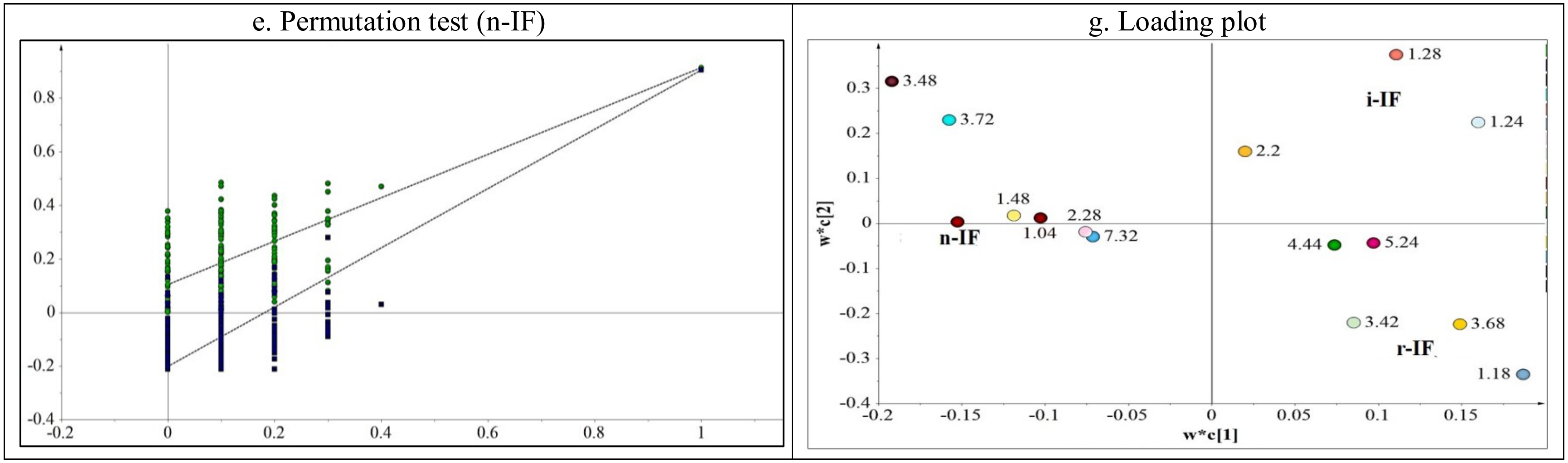

Figure S1. The PCA-X scatter plot, PLS-DA scatter plot, permutation test, and loading plot of the metabolomics analysis.

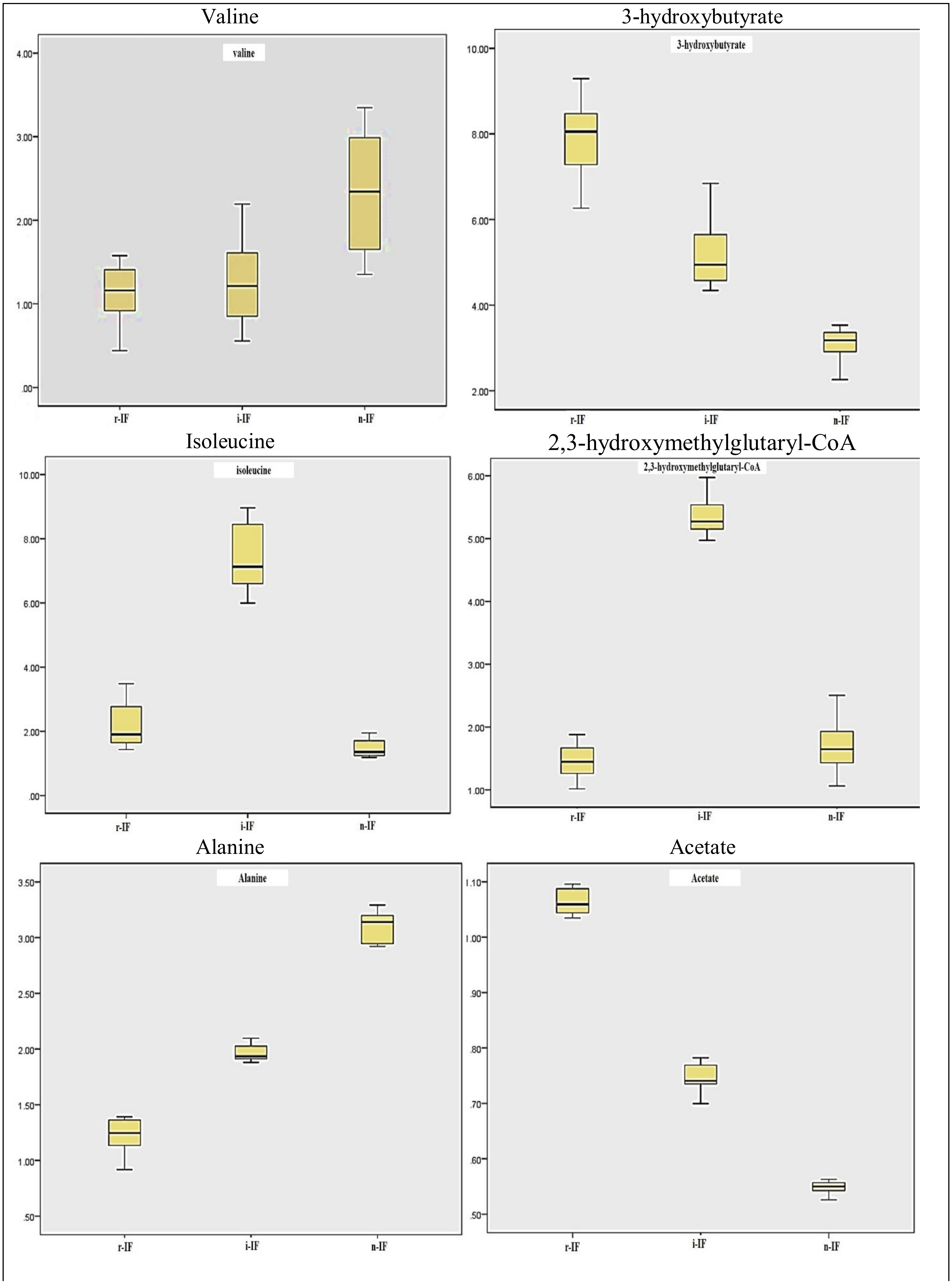

Valine
valine
4.00
3.00
2.00
1.00
.00
r-IF
i-IF
n-IF
3-hydroxybutyrate
3-hydroxybutyrate
10.00
8.00
6.00
4.00
2.00
r-IF
i-IF
n-IF
Isoleucine
isoleucine
10.00
8.00
6.00
4.00
2.00
.00
r-IF
i-IF
n-IF
2,3-hydroxymethylglutaryl-CoA
2,3-hydroxymethylglutaryl-CoA
6.00
5.00
4.00
3.00
2.00
1.00
r-IF
i-IF
n-IF
Alanine
Alanine
3.50
3.00
2.50
2.00
1.50
1.00
.50
r-IF
i-IF
n-IF
Acetate
Acetate
1.10
1.00
.90
.80
.70
.60
.50
r-IF
i-IF
n-IF

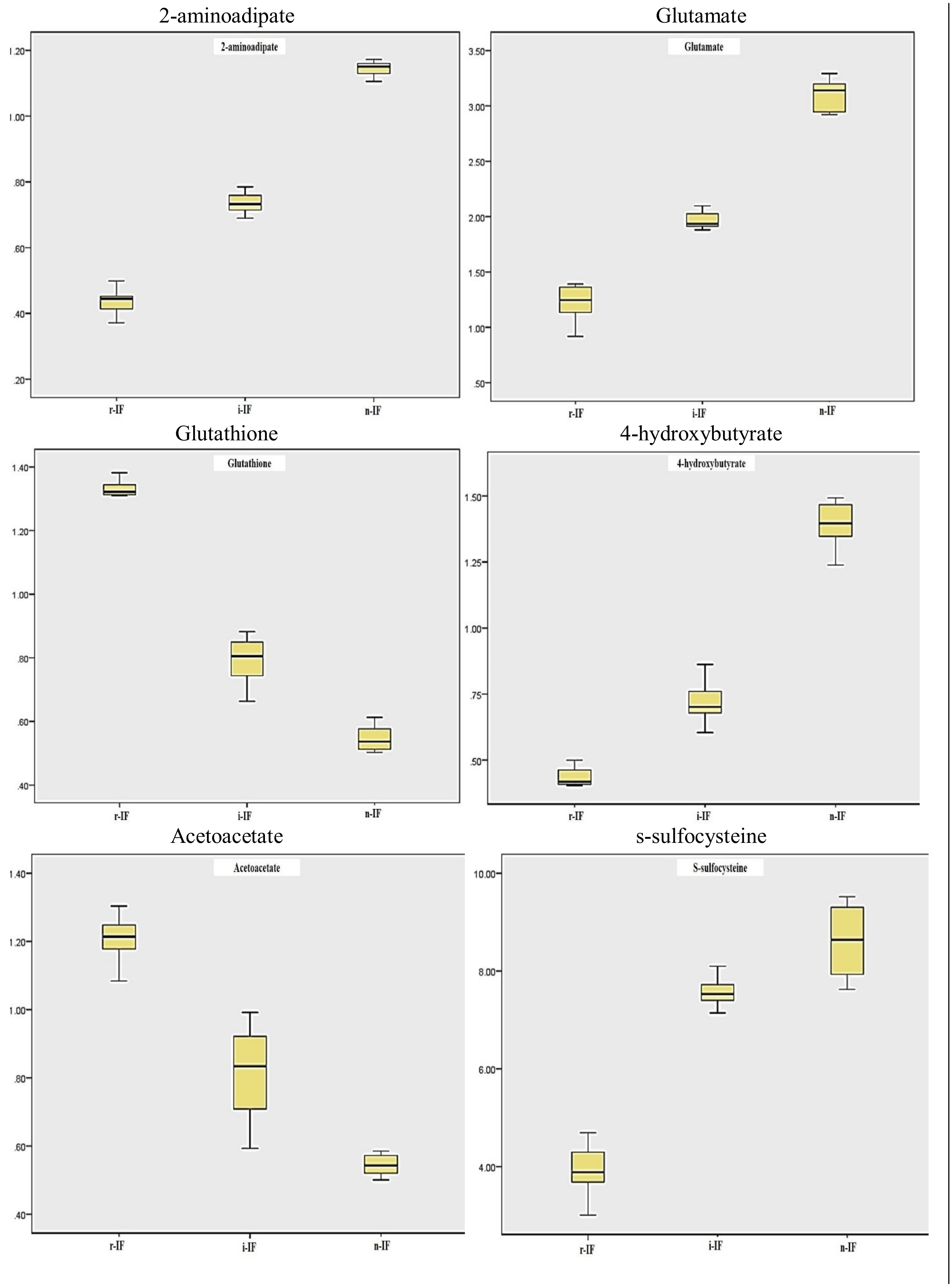

2-aminoadipate
2-aminoadipate
1.20
1.00
.80
.60
.40
.20
r-IF
i-IF
n-IF
Glutamate
Glutamate
3.50
3.00
2.50
2.00
1.50
1.00
.50
r-IF
i-IF
n-IF
Glutathione
Glutathione
1.40
1.20
1.00
.80
.60
.40
r-IF
i-IF
n-IF
4-hydroxybutyrate
4-hydroxybutyrate
1.50
1.25
1.00
.75
.50
r-IF
i-IF
n-IF
Acetoacetate
Acetoacetate
1.40
1.20
1.00
.80
.60
.40
r-IF
i-IF
n-IF
s-sulfocysteine
S-sulfocysteine
10.00
8.00
6.00
4.00
r-IF
i-IF
n-IF

n-acetylglucosamine

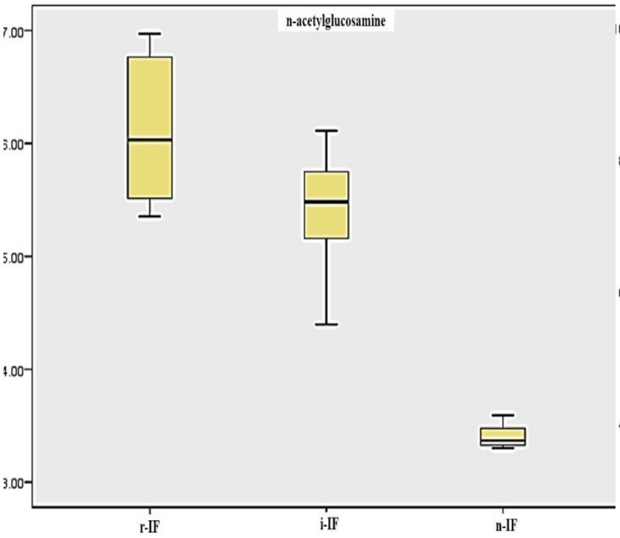

2’-deoxyguanosine

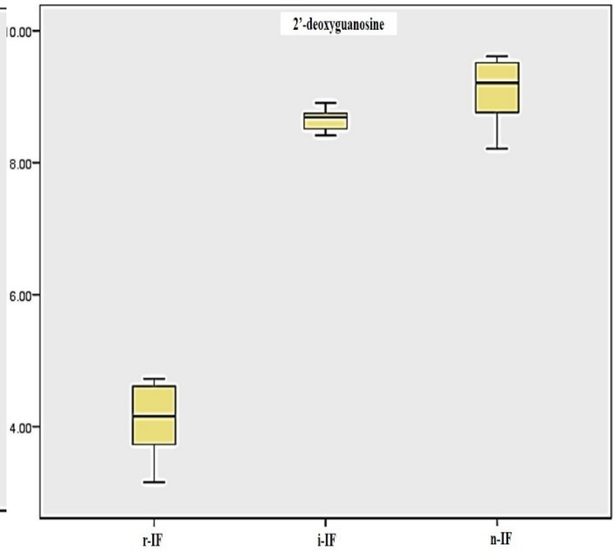

2-hydroxybutyrate

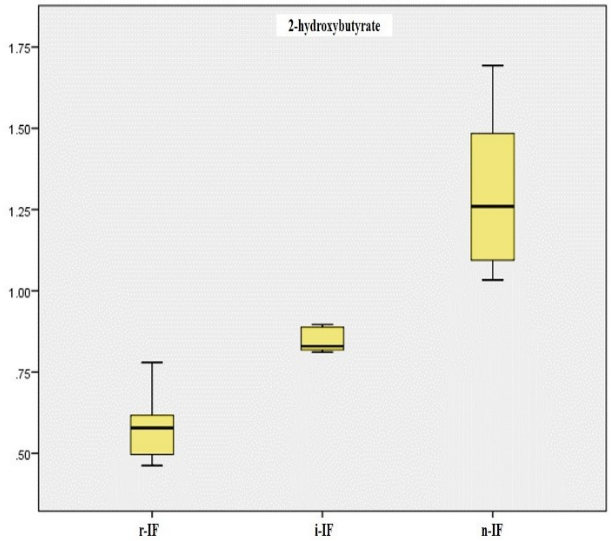

Hippurate

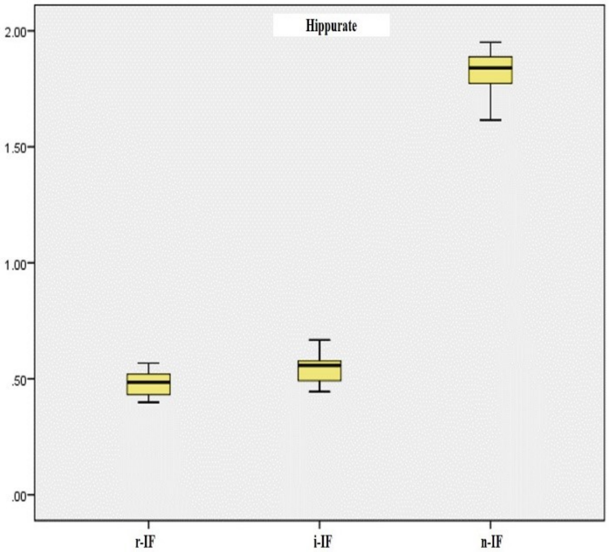

Proline

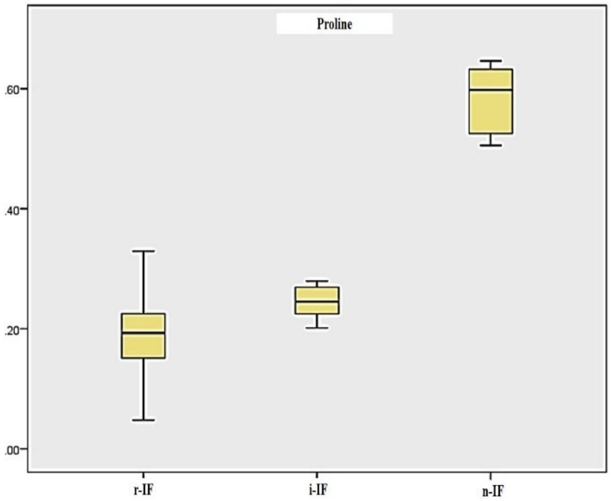

Guanosine

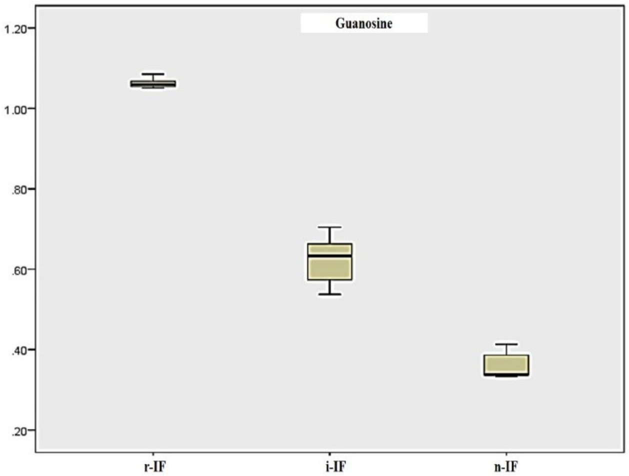

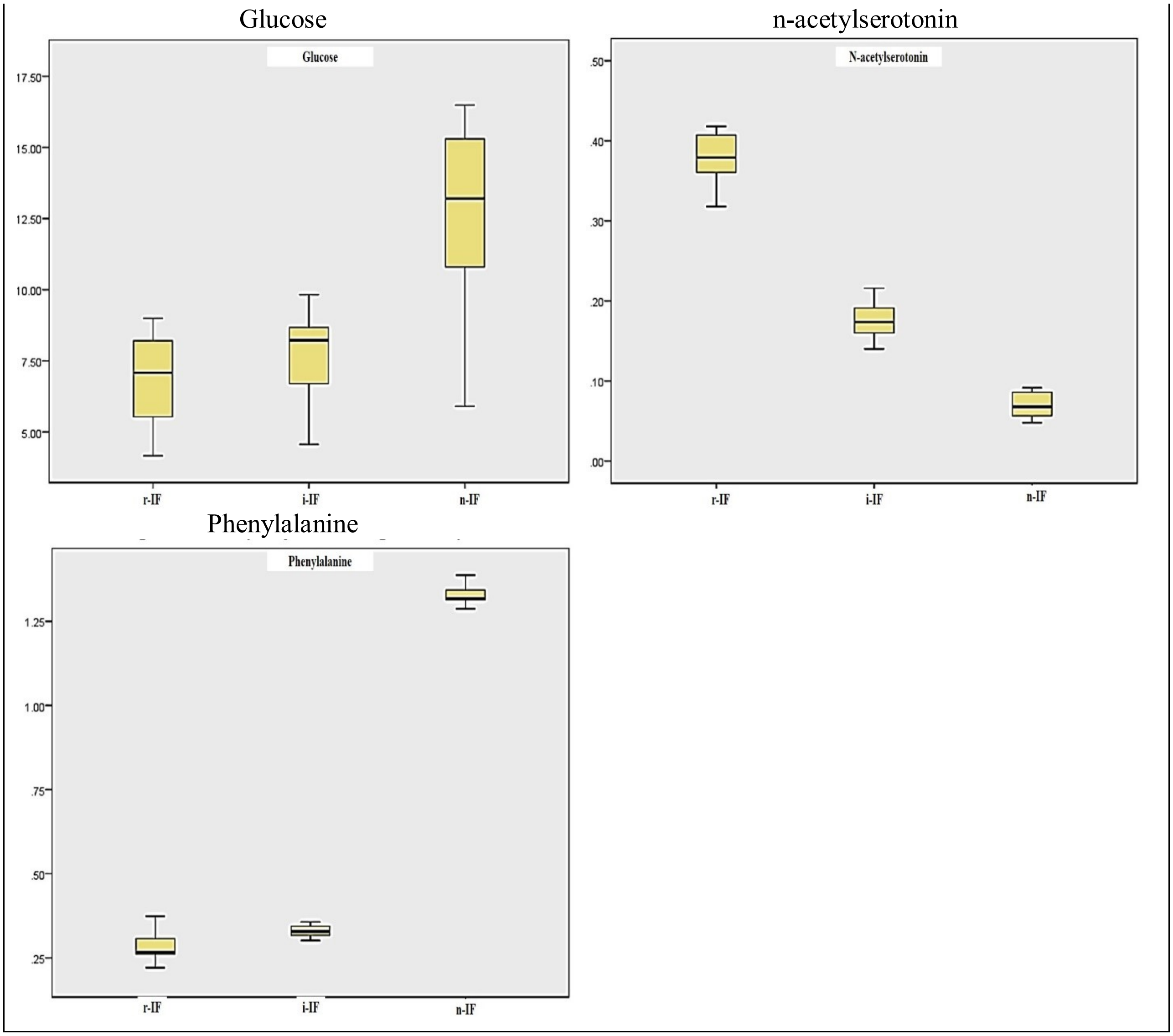

Figure S2. Box-and-whisker plots for each plasma metabolites concentration in relation with fasting status
